# The “Mirror” Reflects

**Published:** 1897-01

**Authors:** 


### The "Miror" Reflects.

The versatile editor of the St. Louis *Medical Mirror* says in the November number: "Speaking of criticism brings to my mind some of our editorial fraternity who are constitutional kickers and ever on the lookout for the weak spots in the armor of their competitors, or I would rather say, colleagues. They never see anything but blemishes or distorted pictures; they seem to be unable to praise anything; I doubt if they would be able to get their consent to praise God, from whom all blessings flow. But don't blame them, they are built that way; they have my sincere sympathy, and that leads me to say that I agree with Ras Wilson, the Philistine, when he says: 'Young man, write as you feel, but try to and feel right. Feel good humored toward every one and every thing. Believe that other folks are just as good, and just as smart as you, for they are. Give 'em your best, and bear in mind that God has sent 'em in his wisdom all the trouble they need, and it's for you to scatter gladness and decent, helpful things as you go. Don't be too particular about how the stuff will look in type, but let 'er go—some one will understand. This is better than to write so dosh bing high and so tarnashun deep that no one understands—let 'er go!' Permit me to say further that while I invite criticism and even enjoy it and try to profit by it, and love commendation, I propose to go on sawing wood, sawing it on my own wood pile, and running the *Medical Mirror* energetically and aggressively without waiting for the consen t of other nations."
—*Journal American Medical Association.*

